# Validation of ICD-9-CM/ICD-10 coding algorithms for the identification of patients with acetaminophen overdose and hepatotoxicity using administrative data

**DOI:** 10.1186/1472-6963-7-159

**Published:** 2007-10-02

**Authors:** Robert P Myers, Yvette Leung, Abdel Aziz M Shaheen, Bing Li

**Affiliations:** 1 Liver Unit, Division of Gastroenterology, Department of Medicine; University of Calgary, Calgary, Alberta, Canada; 2 Department of Community Health Sciences; University of Calgary, Calgary, Alberta, Canada

## Abstract

**Background:**

Acetaminophen overdose is the most common cause of acute liver failure (ALF). Our objective was to develop coding algorithms using administrative data for identifying patients with acetaminophen overdose and hepatic complications.

**Methods:**

Patients hospitalized for acetaminophen overdose were identified using population-based administrative data (1995–2004). Coding algorithms for acetaminophen overdose, hepatotoxicity (alanine aminotransferase >1,000 U/L) and ALF (encephalopathy and international normalized ratio >1.5) were derived using chart abstraction data as the reference and logistic regression analyses.

**Results:**

Of 1,776 potential acetaminophen overdose cases, the charts of 181 patients were reviewed; 139 (77%) had confirmed acetaminophen overdose. An algorithm including codes 965.4 (ICD-9-CM) and T39.1 (ICD-10) was highly accurate (sensitivity 90% [95% confidence interval 84–94%], specificity 83% [69–93%], positive predictive value 95% [89–98%], negative predictive value 71% [57–83%], c-statistic 0.87 [0.80–0.93]). Algorithms for hepatotoxicity (including codes for hepatic necrosis, toxic hepatitis and encephalopathy) and ALF (hepatic necrosis and encephalopathy) were also highly predictive (c-statistics = 0.88). The accuracy of the algorithms was not affected by age, gender, or ICD coding system, but the acetaminophen overdose algorithm varied between hospitals (c-statistics 0.84–0.98; *P *= 0.003).

**Conclusion:**

Administrative databases can be used to identify patients with acetaminophen overdose and hepatic complications. If externally validated, these algorithms will facilitate investigations of the epidemiology and outcomes of acetaminophen overdose.

## Background

Administrative databases are ubiquitous and used in all areas of health care financing and delivery. Health care providers, policy-makers, and payers use administrative data for reimbursement, budgetary planning, monitoring clinical activities, measuring the quality of care, and health services research [1,2]. The critical variable in all of these applications is the patient diagnosis, typically recorded using the *International Classification of Diseases (ICD) Ninth Revision, Clinical Modification *(*ICD-9-CM*) [3] or *Tenth Revision *(*ICD-10*) [4] coding systems. This data can be used to identify specific patient cohorts and assess their clinical outcomes with risk adjustment in many cases. Clearly, the accuracy and completeness of diagnoses within administrative databases is paramount to reaching valid conclusions [5]. As such, the validation of administrative data has been the focus of numerous investigations, typically via audits of medical records. The results of validation studies have varied depending on the type of administrative data (eg. inpatient vs. outpatient and diagnostic vs. procedural), specific disease area and codes used for case identification, and disease severity [5].

To our knowledge, the accuracy of administrative data for the identification of acetaminophen overdose and hepatotoxicity has not been examined. This is surprising considering the magnitude of the problem – acetaminophen overdose is the most common cause of self-deliberate poisoning [6] and acute liver failure (ALF) [7-9]. Recent data from the U.S. ALF Study Group identified acetaminophen as the etiology in approximately 50% of cases [8]. Moreover, numerous studies have examined the epidemiology and outcomes of acetaminophen overdose using administrative data without confirming its accuracy [10-13]. For example, Bateman *et al*. used a nationwide hospital discharge database to demonstrate a reduction in admissions for acetaminophen overdose in Scotland between 1997 and 1999 [10]. This decrease was attributed to package size legislation introduced in the United Kingdom (U.K.) in 1998 due to rising rates of acetaminophen overdose [14-17]. Using similar methodology, Prior *et al*. failed to detect an impact of increased acetaminophen availability on rates of hospitalization for overdose or acute liver toxicity in Canada [11]. To draw valid conclusions from this type of study, the accuracy of these diagnoses must be confirmed.

Therefore, the objectives of our study were: 1) to determine the validity of *ICD-9-CM *and *ICD-10 *diagnostic codes for acetaminophen overdose and hepatotoxicity using a population-based, hospitalization database; and 2) to derive accurate coding algorithms for the identification of these diagnoses for use in future studies employing administrative data.

## Methods

### Study setting and data source

This administrative database and chart abstraction study was conducted in the Calgary Health Region (CHR), one of the largest fully integrated, publicly funded health care systems in Canada. The CHR provides virtually all medical and surgical care to approximately 1.2 million residents of Calgary and surrounding communities in southern Alberta. Included within the region are three large, adult, acute-care hospitals in Calgary. Study subjects were identified using regional administrative data that contains a detailed record of all hospital admissions including diagnostic and procedural information. In this database, diagnoses are coded by trained health records nosologists according to the *International Classification of Disease, Ninth Revision, Clinical Modification *(*ICD-9-CM) *[3] for fiscal years 1995–2001 and *ICD-10 *[4] for 2002–2004 (*ICD-10 *codes shown in italics throughout this manuscript). The database has been used to examine the epidemiology,[18,19] outcomes, [20-22] and coding accuracy [23-26] of a variety of medical conditions. The cohort of potential acetaminophen overdose cases was identified by searching for appropriate *ICD-9-CM *and *ICD-10 *codes in either the primary or 15 secondary diagnosis fields (Table 1). Our preliminary search was designed to maximize sensitivity, thus some of the codes are non-specific. For example, several codes (herein referred to as 'non-specific codes') may include overdoses due to other medications such as salicylates and nonsteroidal anti-inflammatory drugs (NSAIDs) (E950.0, *X40, X60, Y10*).

**Table 1:** ICD-9-CM and ICD-10 codes used to identify potential cases of acetaminophen overdose

**Diagnostic Codes**	**Definition**
**ICD-9-CM**	
965.4 ^†^	Poisoning by aromatic analgesics including acetaminophen
E850.4	Accidental poisoning by aromatic analgesics including acetaminophen
E935.4	Adverse effects of therapeutic use of aromatic analgesics including acetaminophen
E950.0 *	Suicide and self-inflicted poisoning by analgesics, antipyretics and antirheumatics
	
**ICD-10**	
*T39.1 *^†^	Poisoning by 4-aminophenol derivatives
X40 *	Accidental poisoning by and exposure to nonopioid analgesics, antipyretics and antirheumatics including acetaminophen
*Y45.5*	Adverse effects of therapeutic use of 4-aminophenol derivatives
X60 *	Intentional self-poisoning by and exposure to nonopioid analgesics, antipyretics and antirheumatics including acetaminophen
Y10 *	Poisoning by and exposure to nonopioid analgesics, antipyretics and antirheumatics, including acetaminophen, of undetermined intent

^† ^Principal acetaminophen overdose codes.

* Non-specific diagnostic codes.

### Validation study

The validation component of the study was designed to develop coding algorithms for the diagnosis of acetaminophen overdose, hepatotoxicity, and ALF. Since liver injury occurs in only a small minority of overdose patients, [27-31] we intentionally over-sampled this group using a highly sensitive algorithm to identify the cohort for chart review. This algorithm included the following diagnostic codes: hepatic necrosis (570, *K71.1*), toxic hepatitis (573.3, *K71.2, K71.6, K71.9*), hepatic encephalopathy (572.2, *K72.0, K72.9*), hepatorenal syndrome (572.4, *K76.7*), jaundice (782.4, *R17*), coagulopathy (286.7, *D68.4, D68.9*), and adult respiratory distress syndrome (ARDS) (582.82, *J80*) [11,12]. We did not search for liver transplant-related codes because the CHR does not have a liver transplant center. All transplants for CHR residents are performed outside of the health region at the University of Alberta in Edmonton, Alberta. A query of the University of Alberta Liver Transplant database revealed that no CHR residents were transplanted for acetaminophen overdose during the study interval [32]. A control group consisting of randomly-selected patients without these codes, matched 1:1 for admission hospital, was also identified. The inpatient medical records of this cohort were reviewed by a trained internist with gastroenterology experience (YL), blinded to administrative data, using a standardized data abstraction form.

The validity of the diagnoses of interest was assessed using predefined criteria. Acetaminophen overdose was defined as the ingestion of > 4 grams within a 24-hour period (the maximum dosage recommended in the product monograph) [8]. Smaller reported ingestions [8] and unquantifiable overdoses (eg. large amounts of acetaminophen reported unequivocally by the patient or family member) were included as cases if hepatotoxicity occurred (see below) and no alternative cause could be implicated or a serum level >10 mg/L (66 umol/L) was found [30]. Acetaminophen hepatotoxicity was defined by an alanine aminotransferase (ALT) > 1,000 U/L [27-31]. ALF was defined by encephalopathy and an international normalized ratio (INR) > 1.5 [7,8].

### Statistical methods

Data were analyzed using Stata 8.2 software (StataCorp, College Station, TX). The generation of the diagnostic algorithms was performed in a step-wise fashion. First, an algorithm for acetaminophen overdose was generated in the entire cohort. Subsequently, data from these presumed acetaminophen overdose cases was used to generate algorithms for hepatotoxicity and ALF. These algorithms were derived using multivariate logistic regression analyses including the most predictive diagnostic codes for these outcomes. Algorithms were compared using areas under receiver operating characteristics curves (c-statistics) and the non-parametric method of DeLong *et al *[33]. The c-statistic ranges from 0 to 1.0, with 1.0 indicating perfect prediction and 0.5 indicating prediction due to chance alone. C-statistics between 0.7 and 0.8 are generally considered acceptable, while those over 0.8 are considered most desirable. We also calculated sensitivities, specificities, and positive (PPV) and negative predictive values (NPV) for these algorithms, including exact binomial confidence intervals (CI). Because the coding system changed from *ICD-9-CM *to *ICD-10 *in fiscal year 2002, the impact of the study interval (1995–2001 vs. 2002–2004) on the performance of the algorithms was assessed in sensitivity analyses. We also examined patient gender, age (≤ versus > the median), and hospital of admission (to account for different coders) as potential predictors of coding accuracy.

The study protocol was approved by the Conjoint Health Research Ethics Board at the University of Calgary.

## Results

### Diagnosis of acetaminophen overdose

Between fiscal years 1995 and 2004, a total of 1,776 patients met our criteria for potential acetaminophen overdose. Of these, 92 patients were identified by the administrative data as potential cases of acetaminophen hepatotoxicity, and a random sample (n = 92) of the remaining 1,684 patients was selected as controls. The charts of 3 patients were missing, leaving 181 available for data abstraction. The median age of the cohort was 39 years (range 15–84) and 68% were female. After detailed chart review, acetaminophen overdose was confirmed in 139 (77%) of these patients. The remaining 42 patients were admitted for miscellaneous overdoses (n = 28: salicylates [n = 14], NSAIDs [n = 4], and other medications [n = 10]), adverse effects of the therapeutic use of other medications (n = 4), and unrelated diagnoses (n = 10). In 26 of these 42 cases (62%), non-specific codes (ie. potentially coding for medications other than acetaminophen) were recorded.

The operating characteristics of various coding algorithms for the identification of acetaminophen overdose are listed in Table 2. Based on the c-statistics, the optimal algorithm included the principal diagnostic codes for acetaminophen overdose (965.4,*T39.1*) in either the primary or secondary diagnosis fields. This algorithm would detect 132 potential cases, including 125 of the 139 patients with confirmed acetaminophen overdose (sensitivity 90%; 95% CI 84–94%). The specificity, PPV, and NPV for this algorithm were 83% (95% CI 69–93%), 95% (89–98%), and 71% (57–83%), respectively. The operating characteristics were not affected by the diagnostic coding system used (Table 3) and were similar among cases with and without hepatotoxicity as described below (Table 4). Similarly, the c-statistic (0.87 [0.80–0.93]) was not affected by the study interval (ie. 1995–2001: 0.86 [95% CI 0.78–0.94] vs. 2002–2004: 0.88 [0.78–0.97]; *P *= 0.77), patient age (*P *= 0.14) or gender (*P *= 0.33). However, if these codes were limited to the primary diagnosis field (ie. secondary diagnoses excluded), the diagnostic utility decreased substantially (c-statistic 0.72; 95% CI 0.67–0.77; *P *< 0.0005 vs. the algorithm including all diagnosis fields). Moreover, the c-statistic varied between the 3 hospitals included in the study (site 1 [n = 119]: 0.84 [95% CI 0.76–0.92] vs. site 2 [n = 32]: 0.98 [0.94–1.00] vs. site 3 [n = 30]: 0.90 [0.81–0.98]; chi^2 ^[2 df] = 11.46; *P *= 0.003).

**Table 2:** Operating characteristics of diagnostic coding algorithms for the identification of acetaminophen overdose

					**C-statistic (95% CI)**		
					
**Diagnostic Algorithm**	**Sensitivity (95% CI)**	**Specificity (95% CI)**	**PPV (95% CI)**	**NPV (95% CI)**	**All years**	**1994–2001 (ICD-9-CM era)**	**2002–2004 (ICD-10 era)**
All ICD-9-CM and ICD-10 codes *	100% (97–100%)(139/139)	0% (0–8%)(0/42)	77% (70–83%)(139/181)	0% (0/0)	--	--	--
Principal codes (965.4, *T39.1*)	90% (84–94%) (125/139)	83% (69–93%) (35/42)	95% (89–98%) (125/132)	71% (57–83%) (35/49)	0.87 (0.80–0.93) ^‡^	0.86 (0.78–0.94) ^‡^	0.88 (0.78–0.97)
Specific codes (965.4, *T39.1*, E850.4, E935.4, *Y45.5*) ^†^	96% (91–98%) (133/139)	55% (39–70%) (23/42)	88% (81–92%) (133/152)	79% (60–92%) (23/29)	0.75 (0.67–0.83)	0.71 (0.61–0.81)	0.81 (0.69–0.94)

* See Table 1.

^† ^Non-specific codes (ie potentially including overdoses due to medications other than acetaminophen) excluded.

^‡ ^*P *< 0.005 vs. the algorithm limited to specific codes.

CI, confidence interval; NPV, negative predictive value; PPV, positive predictive value.

**Table 3:** Operating characteristics of the coding algorithms according to diagnostic coding system (ICD-9-CM vs. ICD-10) *

**Outcome/Coding System**	**Sensitivity (95% CI)**	**Specificity (95% CI)**	**PPV (95% CI)**	**NPV (95% CI)**	**C-statistic (95% CI)**
**Acetaminophen overdose**					
ICD-9-CM	91% (83–96%) (81/89)	81% (61–93%) (21/26)	94% (87–98%) (81/86)	72% (53–87%) (21/29)	0.86 (0.78–0.94)
ICD-10	88% (77–95%) (44/50)	88% (62–98%) (14/16)	96% (85–99%) (44/46)	70% (46–88%) (14/20)	0.88 (0.78–0.97)
**Hepatotoxicity**					
ICD-9-CM	97% (86–100%) (36/37)	80% (66–90%) (39/49)	78% (64–89%) (36/46)	98% (87–100%) (39/40)	0.88 (0.82–0.95)
ICD-10	86% (65–97%) (19/22)	96% (79–100%) (23/24)	95% (75–100%) (19/20)	88% (70–98%) (23/26)	0.91 (0.83–0.99)
**Acute liver failure**					
ICD-9-CM	85% (55–98%) (11/13)	85% (75–92%) (62/73)	50% (28–72%) (11/22)	97% (89–100%) (62/64)	0.85 (0.74–0.96)
ICD-10	100% (N/A) (9/9)	86% (71–95%) (32/37)	64% (35–87%) (9/14)	100% (NA) (32/32)	0.93 (0.88–0.99)

* None of the comparisons between the ICD-9-CM and ICD-10 coding systems are statistically significant (*P *> 0.05 for all).

CI, confidence interval; NPV, negative predictive value; NA, not applicable; PPV, positive predictive value.

**Table 4:** Operating characteristics of the acetaminophen overdose algorithm according to the development of hepatotoxicity

	**Sensitivity (95% CI)**	**Specificity (95% CI)**	**PPV (95% CI)**	**NPV (95% CI)**	**C-statistic (95% CI)**
**No hepatotoxicity (n = 112)**					
Overall	94% (86–98%) (66/70)	83% (69–93%) (35/42)	90% (81–96%) (66/73)	90% (76–97%) (35/39)	0.89 (0.82–0.95)
ICD-9-CM	94% (82–99%) (44/47)	81% (61–93%) (21/26)	90% (78–97%) (44/49)	88% (68–97%) (21/24)	0.87 (0.79–0.96)
ICD-10	96% (78–100%) (22/23)	88% (62–98%) (14/16)	92% (73–99%) (22/24)	93% (68–100%) (14/15)	0.92 (0.82–1.00)
Hepatotoxicity (n = 69) *					
Overall	86% (75–93%) (59/69)	N/A	100% (NA) (59/59)	0% (NA) (0/10)	N/A
ICD-9-CM	88% (74–96%) (37/42)	N/A	100% (NA) (37/37)	0% (NA) (0/5)	N/A
ICD-10	81% (62–94%) (22/27)	N/A	100% (NA) (22/22)	0% (NA) (0/5)	N/A

* Hepatotoxicity as defined by the following diagnostic codes: hepatic necrosis (570, *K71.1*), toxic hepatitis (573.3, *K71.2*, *K71.6*, *K71.9*), and hepatic encephalopathy (572.2, *K72.0, K72.9*). Because hepatotoxicity cases had acetaminophen overdose by definition, the PPV of the overdose algorithm is 100% in these cases. For the same reason, the specificities and c-statistics cannot be calculated, and the NPVs are 0%.

CI, confidence interval; NPV, negative predictive value; NA, not applicable; PPV, positive predictive value.

### Diagnosis of acetaminophen hepatotoxicity

According to medical record review, 59 of the 132 patients identified by the optimal acetaminophen overdose algorithm developed hepatotoxicity. Table 5 illustrates the frequency of various diagnostic codes consistent with liver injury in these patients versus those not developing hepatotoxicity (n = 73, including 7 patients falsely identified as cases of acetaminophen overdose). Codes for jaundice and hepatorenal syndrome were not recorded in any patient. The most discriminative codes were hepatic necrosis (odds ratio [OR] 17.16; 95% CI 4.65–92.85), toxic hepatitis (OR 7.85; 3.01–21.98), and hepatic encephalopathy (OR 12.96; 1.67–575.6). A diagnostic algorithm including at least one of these codes was highly predictive of hepatotoxicity (OR 77.5; 95% CI 23.3–257.5). The sensitivity, specificity, PPV, and NPV of this algorithm were 93% (95% CI 84–98% [55/59 cases]), 85% (75–92% [62/73]), 83% (72–91% [55/66]), and 94% (85–98% [62/66]), respectively. These figures were not affected by the diagnostic coding system used (Table 3). The corresponding c-statistic (0.89 [95% CI 0.84–0.94]) was not affected by the study interval (*P *= 0.62), patient age (*P *= 0.93), gender (*P *= 0.67), or admission hospital (*P *= 0.89). Moreover, if the algorithm was applied to the entire study cohort of 181 patients (ie. including non-acetaminophen overdose cases), the c-statistic did not change substantially (0.94; 95% CI 0.88–0.99; *P *= 0.24 vs. the primary analysis).

**Table 5:** Frequency of liver-related diagnostic codes according to the development of hepatotoxicity *

**Clinical Diagnosis (Codes)**	**Hepatotoxicity (n = 59)**	**No Hepatotoxicity (n = 73)**	**Odds Ratio (95% CI)**	***P*-value**
Hepatic necrosis (570, *K71.1*)	42.4% (25)	4.1% (3)	17.16 (4.65–92.85)	< 0.0005
Toxic hepatitis (573.3, *K71.2, K71.6, K71.9*)	48.2% (29)	11.0% (8)	7.85 (3.01–21.98)	< 0.0005
Hepatic encephalopathy (572.2, *K72.0, K72.9*)	15.3% (9)	1.4% (1)	12.96 (1.67–575.6)	0.005
Coagulopathy (286.7, *D68.4, D68.9*)	6.8% (4)	4.1% (3)	1.70 (0.27–12.02)	0.70
ARDS (582.82, *J80*)	1.7% (1)	0%	--	0.45

* In patients identified by the acetaminophen overdose coding algorithm including codes 965.4 and *T39.1*. Codes for jaundice and hepatorenal syndrome were not recorded in any patient.

ARDS, adult respiratory distress syndrome. CI, confidence interval.

### Diagnosis of ALF

Of the 132 patients identified as having acetaminophen overdose, 22 developed ALF. The frequencies with which liver-related diagnostic codes were recorded in these patients are listed in Table 6. The most discriminative codes were hepatic encephalopathy (OR 75.46; 95% CI 8.73–3335) and necrosis (OR 9.15; 2.96–28.46). Toxic hepatitis was recorded in fewer patients with ALF than non-ALF cases (18% vs. 30%), but the difference was not significant (*P *= 0.31). A diagnostic algorithm including at least one of the codes for either hepatic encephalopathy or necrosis was highly predictive of ALF (OR 58.8; 95% CI 12.5–276.0). The sensitivity, specificity, PPV, and NPV of this algorithm were 91% (95% CI 71–99% [20/22 cases]), 85% (77–91% [94/110]), 56% (38–72% [20/36]), and 98% (93–100% [94/96]), respectively. These operating characteristics were not affected by the diagnostic coding system used (Table 3). The corresponding c-statistic (0.88 [95% CI 0.81–0.95]), was not affected by the study interval (*P *= 0.18), patient age (*P *= 0.40), gender (*P *= 0.48), admission hospital (*P *= 0.19), or inclusion of patients not identified as overdose cases using the overdose algorithm (*P *= 0.46). If use of the ALF algorithm was restricted to patients identified as having acetaminophen hepatotoxicity, the sensitivity, specificity, PPV, and NPV were 89% (95% CI 67–97% [17/19 cases]), 63% (46–77% [25/40]), 53% (35–71% [17/32]), and 93% (76–99% [25/27]), respectively.

**Table 6:** Frequency of liver-related diagnostic codes according to the development of acute liver failure (ALF) *

**Clinical Diagnosis (Codes)**	**ALF (n = 22)**	**No ALF (n = 110)**	**Odds Ratio (95% CI)**	***P*-value**
Hepatic necrosis (570, *K71.1*)	59.1% (13)	13.6% (15)	9.15 (2.96–28.46)	< 0.0005
Toxic hepatitis (573.3, *K71.2, K71.6, K71.9*)	18.2% (4)	30.0% (33)	0.52 (0.12–1.75)	0.31
Hepatic encephalopathy (572.2, *K72.0, K72.9*)	40.9% (9)	0.9% (1)	75.46 (8.73–3335)	< 0.0005
Coagulopathy (286.7, *D68.4, D68.9*)	9.1% (2)	4.6% (5)	2.10 (0.19–13.88)	0.33
ARDS (582.82, *J80*)	4.6% (1)	0%	--	0.17

* In patients identified by the acetaminophen overdose coding algorithm including codes 965.4 and *T39.1*. Codes for jaundice and hepatorenal syndrome were not recorded in any patient.

ARDS, adult respiratory distress syndrome. CI, confidence interval.

## Discussion

To our knowledge, this is the first study to systematically evaluate the diagnostic accuracy of administrative data for the identification of patients with acetaminophen overdose and liver-related complications. Using a population-based, hospitalization database, we determined that the principal diagnostic codes for acetaminophen overdose (*ICD-9-CM*, 965.4; *ICD-10, T39.1*) had the optimal operating characteristics for case identification. This algorithm was highly sensitive (90%) and specific (83%), thus, the corresponding c-statistic was excellent (0.87). Moreover, the PPV, which reflects the likelihood that an individual truly has the disease for which the administrative data are considered a surrogate, was very high (95%). This algorithm proved much more accurate than two other algorithms which were more sensitive (96–100%), but lacked specificity (0–55%). For example, the non-specific algorithm used for the initial selection of the study cohort identified 181 potential cases, of which only 139 had acetaminophen overdose (PPV, 77%). Of the 42 false positive cases, approximately three-quarters had overdosed or had toxic effects of other medications, frequently salicylates or NSAIDS. Many of these patients were falsely identified by non-specific codes including external causes of injury codes ('E codes'). These codes are frequently used to define the intent of an overdose (eg. intentional, unintentional, homicidal) [34,35] and some include overdoses due to other nonopioid analgesics, antipyretics and antirheumatics (Table 1). Based on these findings, we would advise against using these non-specific codes for the identification of patients with acetaminophen overdose.

In light of our findings, the results of previous and future investigations of acetaminophen overdose should be considered. In the only other study which utilized medical record review to validate acetaminophen overdose diagnoses made using administrative data,[31] the PPV of our optimal algorithm (965.4, *T39.1*) was lower (78%) than observed in our study (95%). Although differences in the study populations and coding accuracy between centers may be involved, we suspect that this difference reflects the inclusion of only patients with single acute ingestions in the latter study,[31] whereas we included patients with multiple time-point ingestions (eg. unintentional overdoses). These patients should not be ignored since they have been linked with a higher frequency of hepatotoxicity and ALF [8,30,36].

In another epidemiologic study from Scotland,[10] Bateman *et al*. used the same codes to report annual hospitalization rates of 91–120 per 100,000 population between 1990 and 1999. Based on the 90% sensitivity that we observed for this algorithm, these figures would appear to underestimate the true incidence of acetaminophen overdose by approximately 10%. Although not a major discrepancy, particularly for the examination of temporal trends, this factor should be considered in other contexts such as studies of health resource utilization and the cost-effectiveness of preventive measures. In the Canadian study by Prior *et al*. [11] and in an analysis of U.S. hospitalization rates by investigators at the Food and Drug Administration (FDA), cases were identified using *ICD-9-CM *codes 965.4 and E850.4. Based on our findings, one might expect the use of the latter E code to have overestimated hospitalization rates. Interestingly, however, our data suggest that the isolated use of this E code does not decrease the accuracy of the algorithm because the principal code 965.4 was recorded in all patients with E850.4 in our database (data not shown).

The algorithms that we have developed hold promise for use in future studies in this field. Specifically, epidemiologic studies evaluating temporal trends and risk factors for acetaminophen overdose will be facilitated. For example, changes in labeling requirements for acetaminophen-containing products were recently recommended (but not acted upon) by an FDA committee in an attempt to reduce the frequency of unintentional overdoses [9,37]. Our algorithm will assist in determining the effectiveness of such recommendations, not only in terms of the number of acetaminophen overdoses, but also the frequency of severe cases associated with hepatotoxicity. Studies from the U.K. have suggested that package size restrictions have reduced the number of severe cases, but these studies are limited by the analysis of data predominantly from transplant centers [14-17]. The algorithms that we have derived can be used with population-based, administrative datasets to overcome the referral bias inherent in the aforementioned studies. Other potential applications include their use to monitor changes in outcomes of acetaminophen overdose as new therapies are developed, and the assessment of controversial risk factors including alcohol abuse and underlying liver disease on rates of hepatotoxicity.

In addition to identifying overdose cases, we aimed to develop accurate coding algorithms for acetaminophen-related liver injury. Although hepatotoxicity occurs in a small minority of cases, acetaminophen overdose is the most common cause of ALF,[7,8] largely due to the magnitude of this problem. According to FDA estimates, nearly 500 deaths attributable to acetaminophen overdose occur annually in the U.S. alone [13]. For the identification of acetaminophen-related hepatotoxicity, defined as an ALT > 1,000 U/L, an algorithm including codes for hepatic necrosis, toxic hepatitis, and hepatic encephalopathy (Table 3) was highly predictive. Specifically, the sensitivity, specificity, and c-statistic of this algorithm were 93%, 85%, and 0.89, respectively. Similar figures were obtained for an ALF algorithm including codes for hepatic necrosis and encephalopathy (Table 5) (sensitivity 91%, specificity 85%, c-statistic 0.88). Although the NPVs of these algorithms were both very high (94–98%), the PPV of the ALF algorithm was lower (56% vs. 83% for the hepatotoxicity algorithm) likely due to the infrequent occurrence of this severe complication. This algorithm cannot accurately identify the subset of hepatotoxicity patients who progress to ALF, presumably due to the overlapping codes used in these algorithms (ie. both include hepatic necrosis and encephalopathy, while the hepatotoxicity algorithm also includes toxic hepatitis). Therefore, the ALF algorithm is most applicable when used in an entire overdose cohort. Interestingly, diagnostic codes for other liver-related complications were either not discriminative (eg. coagulopathy) or not recorded (eg. jaundice and hepatorenal syndrome), arguing against their use for case identification.

In addition to developing these coding algorithms, we performed sensitivity analyses to examine the impact of various patient and system-related factors on their accuracy. Although acetaminophen overdose is more common in younger individuals and females,[10,13] age and gender were not significant in these analyses. Similarly, despite a switch from the *ICD-9-CM *to *ICD-10 *coding system in 2002, the study interval did not affect the operating characteristics. On the contrary, the accuracy of the overdose algorithm varied between three hospitals, although the c-statistics were very good at all facilities (0.84, 0.90 and 0.98, respectively). Nevertheless, the observed differences reinforce the necessity of studies externally validating our findings. Finally, we found that the acetaminophen overdose algorithm was much less sensitive if the appropriate codes were restricted to the primary diagnosis field only. Many of the cases that would have been missed by such an approach had liver-related codes as the main indication for hospitalization.

Our study has several limitations that must be acknowledged. First, we examined a single hospitalization database from a Canadian health region. Since we cannot exclude a regional bias in the coding of administrative data, our findings must be validated using alternative databases (eg. outpatient and emergency databases) in different settings. Second, our chart validation used as the reference standard may be subject to misclassification bias. We minimized this by using a structured data collection instrument and a reviewer of medical records that was blinded to administrative data. Moreover, this reviewer is a trained internist with experience managing patients with acetaminophen overdose and liver disease of all etiologies. Finally, patients with acetaminophen-related hepatotoxicity were over-represented in our study cohort due to our method of patient selection (ie. intentional over-selection due to the relative rarity of this outcome). This factor may have overestimated the PPVs for the liver-related outcomes due to the higher than normal prevalence of these complications in the cohort. Nevertheless, the other measures of diagnostic accuracy that we considered, including the c-statistic, are not affected by disease prevalence.

## Conclusion

In summary, we have validated a hospitalization database for the identification of patients with acetaminophen overdose and associated hepatotoxicity. Although our findings must be validated, this is an important prerequisite for the use of administrative databases in future epidemiologic studies of this common and potentially deadly condition.

## Abbreviations

ALF, acute liver failure; ARDS, adult respiratory distress syndrome; CHR, Calgary Health Region; CI, confidence interval; ICD, International Classification of Diseases; NPV, negative predictive value; NSAIDS, nonsteroidal anti-inflammatory drugs; OR, odds ratio; PPV, positive predictive value

## Competing interests

The author(s) declare that they have no competing interests.

## Authors' contributions

RPM conceived the study, performed statistical analyses, and drafted the manuscript. YL extracted data from medical records and assisted in drafting the manuscript. AMS assisted with statistical analyses and helped draft the manuscript. BL extracted administrative data and assisted in drafting the manuscript. All authors have approved the final version of the manuscript. RPM is the guarantor.

## Pre-publication history

The pre-publication history for this paper can be accessed here:


